# Conservative Management of the Duodenal Injury during Percutaneous Nephrostomy Placement: A Few and Far between Complications of the Urological Literature

**DOI:** 10.1155/2021/8221488

**Published:** 2021-12-31

**Authors:** Aykut Colakerol, Mustafa Zafer Temiz, Mubarek Bargicho Adem, Kamil Ozdogan, Fatih Celebi, Engin Kandirali, Ahmet Yaser Muslumanoglu

**Affiliations:** ^1^Department of Urology, Bagcilar Training and Research Hospital, Istanbul, Turkey; ^2^Department of General Surgery, Urology Unit, St. Paul's Hospital Millennium Medical College, Addis Ababa, Ethiopia; ^3^Department of General Surgery, Bagcilar Training and Research Hospital, Istanbul, Turkey

## Abstract

Herein, we reported a duodenal perforation case as an intestinal injury during a percutaneous nephrostomy procedure. A 73-year-old woman with bilateral nephrostomy catheters was applied to the emergency service with right flank pain. Early in the day, her bilateral nephrostomy catheters had been changed. On physical examination, she had a defense and rebound at her right quadrant, and costovertebral angle tenderness was also positive. In the contrast-enhanced abdominal computed tomography scan, the right nephrostomy catheter was located in the second part of the duodenum, and the contrast agent did not leak into the peritoneum from the injury area. We decided on conservative management of the case with active surveillance using daily blood tests and physical examinations. The nephrostomy catheter in the duodenum was left to prevent fistula between the duodenum and the skin, and a new one was placed in the right kidney. The broad spectrum antibiotherapy regime was applied, and the patient was followed up closely. The catheter in the duodenum was removed on the 20th day, uneventfully, and the patient was discharged successfully on the 24th day with her permanent bilateral nephrostomy tubes. On the first follow-up, one month later, the patient had no active medical complaint.

## 1. Introduction

Percutaneous nephrostomy (PCN) is an interventional method of accessing the renal collecting system through the skin. It is a commonly used treatment approach in patients with urinary obstruction, especially when ureteral stent replacement is not possible [1, 2]. During the procedure, some major and minor complications can occur [1, 2]. Sepsis and massive bleeding are the most common major complications associated with PCN [2]. Intestinal complications are commonly associated with percutaneous nephrolithotomy, and to our knowledge, there is only one case with an intestinal complication in the form of duodenal perforation during PCN procedure without renal stone fragmentation [3]. Herein, after obtaining written informed patient consent, we report the second case of duodenal perforation as an intestinal injury during PCN.

## 2. Case Report

**Figure 1:**
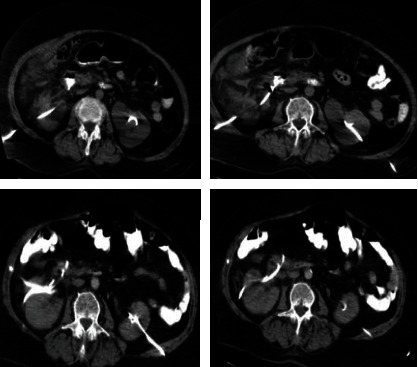
Oral and intravenous contrast-enhanced abdominal CT images. The right nephrostomy tube was located in the second part of the duodenum, and there was no leakage of contrast substance to the retroperitoneal site.

A 73-year-old woman with bilateral nephrostomy catheters was applied to the emergency service with right flank pain that started 10 hours prior after bilateral nephrostomy catheter replacement. She had a history of chronic kidney failure. She had been followed up with bilateral nephrostomy catheters for four years due to the bilateral ureteral stricture secondary to stone disease. Early in the day, her bilateral nephrostomy catheters had been changed under guiding assistance from ultrasound imaging. New nephrostomy placement was performed using the current tube tract instead of fresh stick/new site of nephrostomy tube placement. During the procedure, no signs of complication and unexpected events were recorded, and a day case hospitalization was provided for routine postprocedural monitoring. A few hours later, during physical examination, she had a defense and rebound at her right quadrant, and costovertebral angle tenderness was also positive. Body temperature and blood pressure were 36.7°C and 130/70 mmHg. Serum creatinine, hemoglobin, and C-reactive protein (CRP) levels were 2.43 mg/dL, 11.6 g/dL, and 90 mg/L, respectively. White blood cell count (WBC) was 14.9 × 10^3^u/L. In the right nephrostomy bag, there was 800 cc fluid compatible with bilious fluid and small intestinal content. Subsequently, the patient underwent noncontrast-enhanced abdominal computed tomography (CT) scan to confirm the nephrostomy catheter placements. CT scan showed that the right nephrostomy catheter was located in the second part of the duodenum by piercing the right kidney. The patient's oral intake had been stopped, intravenous hydration and 2 × 1000 mg meropenem (Meronem, AstraZeneca BioPharma. Co., Macclesfield, UK) therapies had been started, and she consulted to the general surgery department which suggested oral and intravenous abdominal contrast-enhanced CT (CECT) imaging. In the CECT scan, the contrast agent did not leak into the peritoneum from the duodenal injury area (Figure 1). We decided on conservative management of the case with active surveillance using daily blood tests and physical examinations. The nephrostomy catheter was left in the duodenum to prevent fistula formulation between the duodenum and the skin, and a new one was placed in the right kidney.

**Figure 2:**
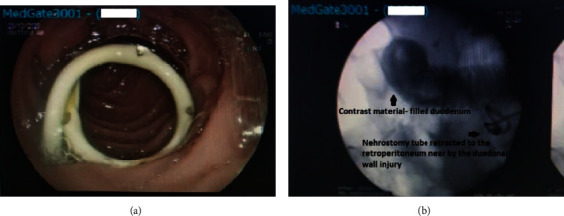
(a) Nephrostomy catheter located in the part of duodenum. (b) During removal of the nephrostomy tube, oral contrast was given, and there was no sign of leakage to the retroperitoneal area.

On the second day, a nasogastric tube was inserted and parenteral nutrition was started. The patient was stable, the creatinine level was increased to 2.7 mg/dL, WBC count was increased to 17.27 × 10^3^ u/L, and CRP level was increased to 184 mg/L. The antibiotherapy regime was rearranged as intravenous injections of 3 × 1000 mg meropenem (Meronem, AstraZeneca BioPharma. Co., Macclesfield, UK), 2 × 1000 mg vancomycin (Vancomycin HCL DBL, Orna Pharma. Co., Istanbul, Turkey), and 3 × 500 mg metronidazole (Flagyl, Sanofi Pharma. Co., Kırklareli, Turkey) per day. Intravenous 1 × 40 mg proton pump inhibitor, pantoprazole (Pantpas, Takeda GmbH, Dingen, Germany), was also administrated daily. Daily outputs from catheters were about 1500 cc and 500 cc for nephrostomy tubes in the renal pelvis and in the duodenum, respectively. All blood parameters showed a gradual decrease during the conservative management. As mentioned above, the duodenal nephrostomy tube was not removed to prevent a new fistula tract formation between the duodenum and the skin during the three weeks. On the 20th day, the duodenal nephrostomy tube drainage was 50 cc. On the same day, the tube was retracted from the duodenal lumen to the retroperitoneal area near by the duodenal wall injury under direct endoscopic visualization in an attempt to avoid the potential retroperitoneal injury from possible leakage (Figure 2(a)). During the three-day follow-up of the retracted tube, no drainage was recorded and the tube was removed. During removal of the duodenal nephrostomy tube, oral contrast was given, and no signs of leakage to the retroperitoneal area were detected on fluoroscopy (Figure 2(b)). The period after the removal of the duodenal nephrostomy tube was uneventful. The serum CRP level exhibited gradual decrease and, finally, decreased to her basal value. Follow-up blood test results during the conservative management of the case are as shown in Table 1. The relatively higher basal CRP value was attributed to her chronic kidney disease because of no other additional signs of acute infection or severe disease. The patient was discharged successfully on the 24th day with her permanent bilateral nephrostomy tubes. On the first follow-up, one month later, the patient had no active medical complaint.

## 3. Discussion

The PCN procedure was firstly described by Goodwin et al. [4] in 1955, and then, it has become one of the most common urological procedures in clinical practice [2]. Because of the image-guided nature of the PCN procedure, commonly, it is smooth sailing. Safety and effectiveness of the procedure has been proven in the literature [5]. Technical success of the PCN is described as 93–99% [6]. Nevertheless, it is not a procedure that is free of complications. The general complication rate of PCN is approximately 10% [6]. Major complications such as septic shock and massive hemorrhage associated with PCN procedures are seen in about 4.5% of the cases [2]. Minor complications including catheter blockage, leaking, fracturing, and kinking of the catheter occur in 6.1% of the cases [2]. Rarely seen complications of the procedure are intestinal, pleural, and renal pelvic injuries with incidences of about 0.1-1% [3, 6].

Percutaneous renal procedures associated with damage to the gastrointestinal tract are extremely rare and most often seen in the colon. Only five cases of duodenal damage associated with percutaneous procedures have been reported so far [7]. Moreover, all of them were specifically associated with percutaneous nephrolithotomy (PCNL), but one. To our knowledge, there is just one duodenal injury case during the PCN procedure, reported in 2019 [2, 3, 6]. Mehdi et al. [3] presented the first PCN-associated duodenal injury in a 45-year-old woman with emphysematous pyelonephritis who underwent PCN procedure. The authors diagnosed the duodenal injury with oral and intravenous abdominal CECT after bilious fluid drainage from the nephrostomy catheter. In the present case, similarly, the diagnosis of duodenal injury was made with CECT scan, after the suspicion of bilious fluid and small intestinal content output from the nephrostomy catheter, within 3 hours. The patient suffered from persistent lower back pain in the right side and vomiting as well.

Data about the management of duodenal injury after percutaneous renal procedures are very limited. With the present case, there are only two and four cases of duodenal injury after PCN and PCNL procedures, respectively, in the urological literature. Previous three cases associated with PCNL were managed with conservative approaches such as perinephric drainage, nasogastric tube, H2 antagonist, and total parenteral nutrition, whereas the other one was treated with laparotomy and primary suture repair [7]. The first case after PCN was similarly managed with open repair of the duodenal perforation [3]. In our case, we preferred the conservative management with parenteral nutrition and nasogastric tube and intravenous proton pump inhibitor and broad spectrum antibiotic administration. The absence of duodenal leakage and early placement of the second nephrostomy enabled the patient to be followed up stably. The conservative approach, instead of laparotomy with additional potential morbidity, turned out to be advantageous in the present case.

## Figures and Tables

**Table 1:** Serum CRP and creatinine levels and WBC count of the patient during the hospitalization. (On the third day, a second nephrostomy tube was replaced, and after 24 days of follow-up with a broad spectrum of antibiotics, the patient was discharged.)

	Serum CRP levels (mg/L)	Serum creatinine levels (mg/dL)	WBC (×10^3^ u/L)
Basal value (before the bilateral nephrostomy catheter replacement and duodenal injury)	48	1.8	—
Day 1	90	2.43	14.9
Day 2	184	2.73	17.2
Day 3	300	2.76	10.0
Day 4	211	2.66	6.6
Day 5	107	2.25	6.6
Day 7	91	1.94	6.5
Day 24 (discharged)	47	1.8	5.1

CRP: C-reactive protein; WBC: white blood cell count.

## Data Availability

The data used to support the findings of this study are available from the corresponding author upon request.
